# Jumonji/Arid1b (Jarid1b) protein modulates human esophageal cancer cell growth

**DOI:** 10.3892/mco.2013.127

**Published:** 2013-05-21

**Authors:** YOSHIHIRO KANO, MASAMITSU KONNO, KATSUYA OHTA, NAOTSUGU HARAGUCHI, SHIMPEI NISHIKAWA, YOSHINORI KAGAWA, ATSUSHI HAMABE, SHINICHIRO HASEGAWA, HISATAKA OGAWA, TAKAHITO FUKUSUMI, YUKO NOGUCHI, MIYUKI OZAKI, TOSHIHIRO KUDO, DAISUKE SAKAI, TAROH SATOH, MASARU ISHII, EIICHI MIZOHATA, TAKESHI INOUE, MASAKI MORI, YUICHIRO DOKI, HIDESHI ISHII

**Affiliations:** 1 Departments of Frontier Science for Cancer and Chemotherapy, Osaka University, Graduate School of Medicine, Suita, Osaka 565-0871;; 2 Gastroenterological Surgery, Osaka University, Graduate School of Medicine, Suita, Osaka 565-0871;; 3 Otorhinolaryngology-Head and Neck Surgery, Osaka University, Graduate School of Medicine, Suita, Osaka 565-0871;; 4 Laboratory of Cellular Dynamics, WPI-Immunology Frontier Research Center, Osaka University, Suita, Osaka 565-0871;; 5 Japan Science and Technology Agency, Core Research for Evolutional Science and Technology, Chiyoda, Tokyo 102-0075;; 6 Osaka University, Graduate School of Technology, Suita, Osaka 565-0871, Japan

**Keywords:** Jumonji/Arid1B, esophageal cancer, gastrointestinal organs

## Abstract

Although esophageal cancer is highly heterogeneous and the involvement of epigenetic regulation of cancer stem cells is highly suspected, the biological significance of epigenetically modified molecules that regulate different subpopulations remains to be firmly established. Using esophageal cancer cells, we investigated the functional roles of the H3K4 demethylase Jumonji/Arid1b (Jarid1b) (Kdm5b/Plu-1/Rbp2-h1), an epigenetic factor that is required for continuous cell growth in melanoma. *JARID1B* knockdown resulted in the suppression of esophageal cancer cell growth, sphere formation and invasion ability and was associated with loss of epithelial marker expression. However, these inhibitory effects observed on tumor formation were reverted subsequent to subcutaneous inoculation of these cells into immune-deficient mice. These results indicated that *JARID1B* plays a role in maintaining cancer stem cells in the esophagus and justifies the rationale for studying the effects of continuous inhibition of this epigenetic factor in esophageal cancer.

## Introduction

Esophageal cancer is one of the most lethal human cancers that occur worldwide. It is the eighth most common cancer in several European countries and its incidence is on the increase in Western countries (1–3). Barrett’s esophagus, the only known precursor to esophageal adenocarcinoma, is prevalent in Western countries (1–3). In Barrett’s esophagus, a human metaplastic condition is characterized by a posterior intestinal-like phenotype in an anterior organ. Underlying it is a mechanism of epigenetically regulated, developmentally critical genes, such as the *HOXB* family (4). By contrast, the squamous cell carcinoma of the esophagus is predominant in Asia, including Japan (1–3).

A previous study suggested that the genetic and epigenetic alterations, which constrain tumor suppressor genes and activate oncogenes, are involved in the initiation, progression and development of carcinogenesis in the esophagus, which is asssociated with exposure to environmental carcinogens (5). Specifically, animal model analogies of environmental carcinogenesis in humans indicated that alterations in the expression of microRNAs, such as *miR-31* and *miR-21*, characterized epithelial tumor progression in the esophagus. The microRNAs were also detected in circulating blood and were associated with inflammation of the esophagus (6–8).

The most reliable markers currently available for predicting cancer risk are findings of the degree of dysplasia in endoscopic biopsies of the esophagus (9). Although epigenetic regulation is eventually involved in tumor development in the esophagus, few molecular biomarkers have been translated to widespread clinical practice (9). Epigenetic studies have shown that the aberrant DNA methylation of tumor suppressor genes is involved in esophageal cancer, as well as in adenocarcinoma, squamous cell carcinoma and Barrett’s esophagus. In addition, several aberrantly methylated genes have been studied with regard to early detection or as diagnostic markers and for estimating prognosis or predicting responses to treatment (9).

In esophageal cancers, alterations in histone modifications have also been identified. Histone deacetylase inhibitors have been shown to enhance radiation responses through a mechanism accompanied by an increase in the levels of γH2ax, an indicator of double-strand breaks (DSBs), and a decrease in Rad51 expression, a DSB repair protein. This suggests that histone deacetylase inhibitors are safe, promising radiosensitizers for esophageal cancer radiotherapy (10). Nevertheless, the significance of histone modifiers remains to be determined.

Through the use of H3K4 demethylase Jarid1b (Kdm5b/Plu-1/Rbp2-h1) as a biomarker, a small subpopulation of tumor-initiating melanoma cells was isolated. *JARID1B* knockdown ultimately inhibited melanoma cell growth (11). In this study, we investigated the effects of *JARID1B* knockdown on squamous cell carcinoma of the esophagus using lentiviral transfer of small hairpin (sh) RNA molecules for inhibition. Our findings are compatible with the hypothesis that, similar to genetic alterations, epigenetic aberrations including histone modifications significantly contribute to tumor initiation and progression in gastrointestinal cancers. This observation provides a rationale to study the usefulness of *JARID1B* in the diagnosis and therapeutic approaches to esophageal cancer.

## Materials and methods

### Cell culture and transfection

Human esophageal squamous cell carcinoma cell lines (TE4 and TE8) were maintained at 37°C in RPMI-1640 medium supplemented with 10% fetal bovine serum (FBS). For shRNA-mediated knockdown of endogenous *JARID1B*, lentiviruses were purchased from Santa Cruz Biotechnology, Inc. (Santa Cruz, CA, USA). The cells were cultured in 12-well plates. After 24 h, cells were infected with 20 *μ*l/well of shRNA lentivirus particles in the presence of 5 *μ*g/ml polybrene (Sigma-Aldrich, St. Louis, MO, USA). After another 24 h, the culture medium was removed and replaced with 1 ml of complete medium without polybrene. Subsequently, shRNA-infected cells were treated and selected with 2 *μ*g/ml of puromycin (Sigma-Aldrich).

### RNA extraction and real-time quantitative polymerase chain reaction (PCR)

Total RNA was extracted from cells using Qiagen RNeasy mini kits and was reverse-transcribed (RT) into cDNA using High Capacity RNA to cDNA kits (Applied Biosystems, Carlsbad, CA, USA). Samples were analyzed by real-time quantitative RT-PCR (TaqMan Master Mix Kit, Applied Biosystems) to detect the expression of the human genes *JARID1B*, *SNAIL*, *VIMENTIN* and *ACTB*. The primers were used were: *JARID1B,* forward: 5′-GCTTAATGGCAA AAGGCAAAC-3′ and reverse: 5′-CGGAGCTCATTCACT GTCAAC-3′; *SNAIL*, forward: 5′-GCTGCAGGACTCT AATCCAGA-3′ and reverse: 5′-ATCTCCGGAGGTGGG ATG-3′; *VIMENTIN*, forward: 5′-AAAGTGTGGCTGCCAA GAAC-3′ and reverse: 5′-AGCCTCAGAGAGGTCAGCAA-3′; *ACTB*, forward: 5′-AGAGCTACGAGCTGCCTGAC-3′ and reverse: 5′-CGTGGATGCCACAGGACT-3′.

### Proliferation assay

Cell proliferation was determined with the WST-8 assay using Cell Counting kit-8 (Dojindo, Kumamoto, Japan), in which 2,000 cells/well were placed in a 96-well plate. After 24, 48, 72 and 96 h, 10 *μ*l of Cell Counting kit-8 solution [2-(2-methoxy-4-nitrophenyl)-3-(4-nitrophenyl)-5-(2,4-disulfophenyl)-2H-tetrazolium, monosodium salt] was added to each well and incubated for 1 h. Cell viability was determined by reading the optical density in each well at 450 nm.

### Invasion assay

Cancer cell invasion was assessed using 24-well BioCoat™ Matrigel Invasion Chambers (8 *μ*m; Becton-Dickinson, Franklin Lakes, NJ, USA) according to the manufacturer’s protocol. Briefly, 5×10^4^ cells were placed in the top chamber. The bottom chamber contained 10% FBS as a chemoattractant. After 96-h incubation, the non-invasive cells on the upper surface of the membrane were removed with cotton swabs. The cells that adhered to the lower surface of the membrane were fixed and stained using Diff-Quick (Sysmex Internal Reagents Co., Ltd., Kobe, Japan) and the number of cells was counted.

### Animal experiments

A total of 10^2^ or 10^3^ cells (*JARID1B* knockdown TE4 cells and control TE4 cells), mixed with BD matrigel (Becton Dickinson) at a 1:1 ratio, were injected subcutaneously into NOD/SCID mice. These mice were examined for up to 10 weeks and sacrificed when the tumors reached a maximum diameter of 15 mm. The animal studies were approved by the Animal Experiments Committee of Osaka University (Suita, Japan).

## Results

### JARID1B knockdown suppresses esophageal cancer cell growth

**Figure 1.**
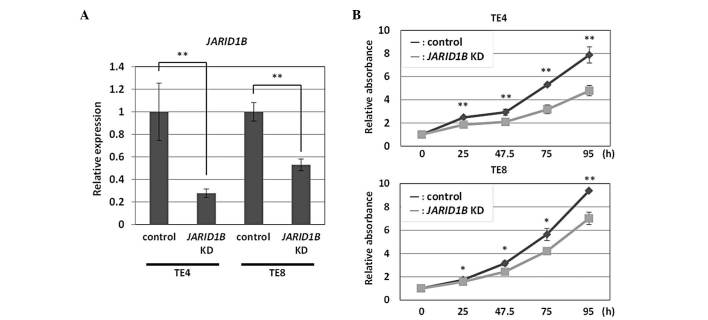
Lentiviral-mediated knockdown of *JARID1B* suppresses esophageal cancer cell growth. (A) Quantitative real-time polymerase chain reaction (RT-PCR) analysis of *JARID1B* mRNA in esophageal cancer TE4 and TE8 cells. Results are relative to control *ACTB* mRNA expression. (B) Cell counting assay for *JARID1B* knockdown (KD) cells. Tumor cell growth was assessed in growth medium at the indicated times. *JARID1B* KD, lentiviral-mediated knockdown of *JARID1B*; control, lentiviral vector transfection. Student’s t-test; *P<0.05 and **P<0.01 vs. control.

A lentiviral vector-mediated shRNA knockdown system was developed for the efficient knockdown of *JARID1B*. Following the introduction of shRNA, TE4 and TE8 esophageal squamous cell carcinoma cells were grown in growth medium to select transfectants. RNA was extracted from these cells and used for quantitative RT-PCR analysis. The transfectants with the *JARID1B* knockdown vector had reduced amounts of endogenous *JARID1B* transcripts as compared with the control vector transfectants for TE4 and TE8 cells (Fig. 1A). Based on cell counts, *JARID1B* knockdown TE4 cells exhibited reduced cell growth during the periods indicated in Fig. 1B. Similar results were obtained with *JARID1B* knockdown TE8 cells (Fig. 1B). These results indicated that *JARID1B* knockdown suppressed esophageal tumor cell growth.

### JARID1B knockdown suppresses esophageal cancer cell invasion

**Figure 2.**
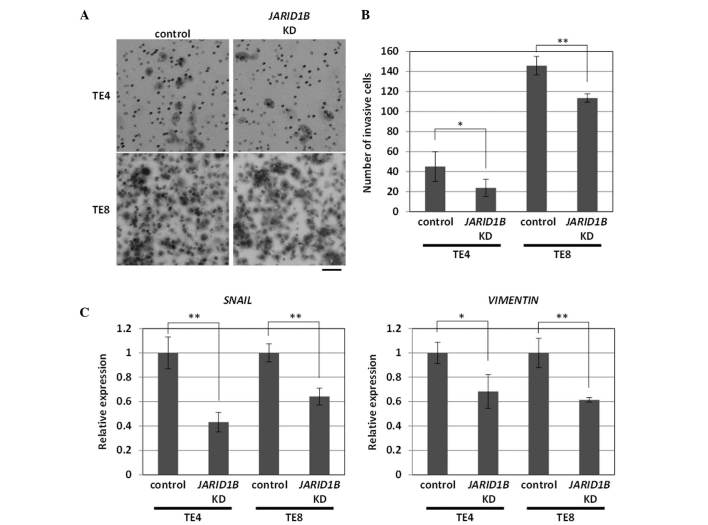
Lentiviral-mediated knockdown (KD) of *JARID1B* suppresses esophageal cancer cell invasion. (A) Cell invasion assay was performed as described in Materials and methods. (B) Invading cells were counted in the chamber slide as indicated. (C) Quantitative real-time polymerase chain reaction (RT-PCR) analysis of *SNAIL* and *VIMENTIN* mRNAs in esophageal cancer TE4 and TE8 cells. Results are relative to control *ACTB* mRNA expression. Scale bar, 100 *μ*m. Student’s t-test; *P<0.05 and **P<0.01 vs. control.

Cancer invasion and metastasis are frequently associated with cancer heterogeneity and are important factors that affect cancer management (12,13). Thus, the control of cancer invasion is crucial. Concomitant with the observed cell growth inhibition, the invasion ability of *JARID1B* knockdown TE4 cells was significantly suppressed (Fig. 2A). Similar results were obtained with *JARID1B* knockdown TE8 cells, although total cell invasion was more apparent than with TE4 cells (Fig. 2A).

To explore the possible underlying mechanisms, we examined the expression of epithelial-mesenchymal transition (EMT) genes, ES-like genes (*SOX2*, *OCT3/4*, *KLF4* and *c-MYC*) for which aggressive phenotypes have been suggested (14) and tumor suppressor genes (*p21/Waf1/Cip1/Sdi1* and *p16/INK4A*). We found reproducible results for the significant inhibition of EMT-related genes, *SNAIL* and *VIMENTIN*, in *JARID1B* knockdown TE4 and TE8 cells (Fig. 2B). Thus, these results indicated that *JARID1B* knockdown reduced tumor cell growth and invasion via the induction of a network of EMT-related genes.

### JARID1B knockdown suppresses esophageal cancer sphere formation

**Figure 3.**
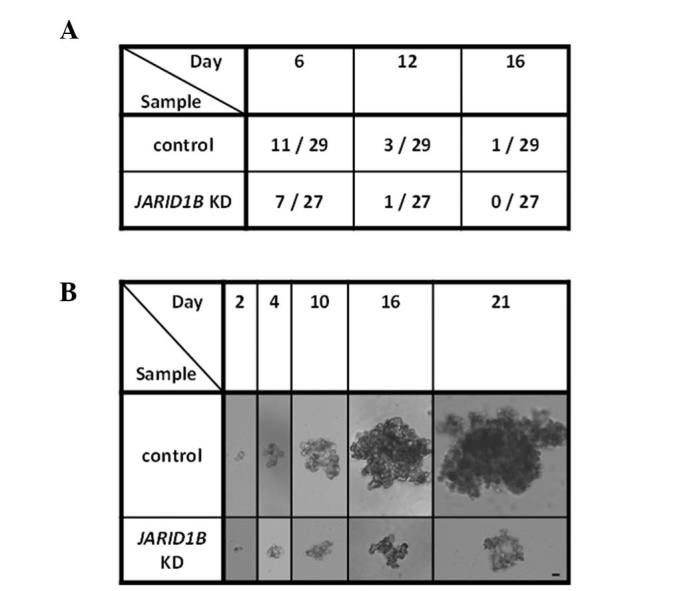
Lentiviral-mediated knockdown (KD) of *JARID1B* suppresses sphere formation of esophageal cancer cells. (A) Sphere formation assay. Spheres of TE4 cells were counted at the indicated times. (B) The phenotype of formed spheres. Photomicrographs of TE4 spheres were captured with a phase contrast microscope. Scale bar, 100 *μ*m.

Concerning the heterogeneity of cells within tumors, the involvement of cancer stem cells has been discussed with regard to self-renewal and re-establishment of tumor tissues (15,16). To assess the self-renewal of cancer cells, TE4 and TE8 *JARID1B* knockdown and control transfectant cells were used in sphere formation assays. *JARID1B* knockdown resulted in the inhibition of sphere formation as observed on days 6, 12 and 16 (representative data shown in Fig. 3A and B). Thus, these results suggest that *JARID1B* knockdown reduced the self-renewal activity of esophageal cancer cells.

### JARID1B knockdown suppresses esophageal cancer tumorigenicity

**Figure 4.**
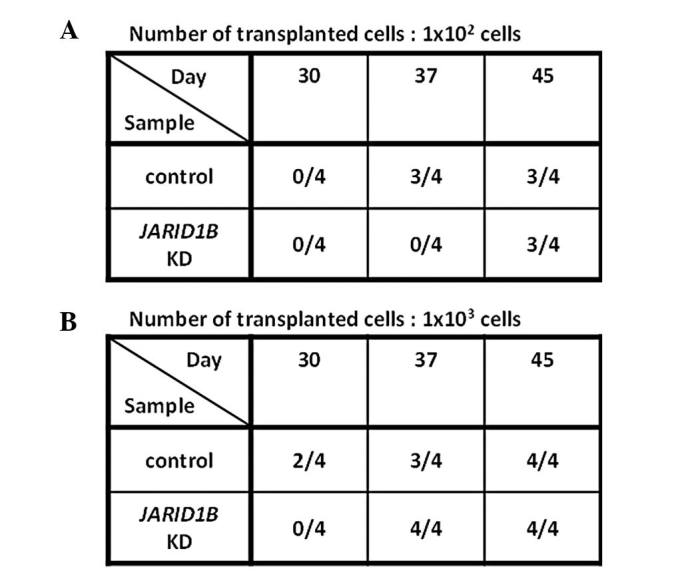
Tumorigenicity following lentiviral-mediated knockdown (KD) of *JARID1B*. To assess tumorigenicity, (A) 10^2^ and (B) 10^3^ cells were subcutaneously inoculated into NOD/SCID mice. The mice in which tumors formed were counted as indicated.

The effects of *JARID1B* knockdown *in vivo* were examined by inoculating *JARID1B* knockdown TE4 and TE8 cells into immune-deficient NOD/SCID mice. When 10^2^ *JARID1B* knockdown TE4 or TE8 cells were inoculated subcutaneously into mice, tumorigenicity was reduced as observed on days 30 and 37 (representative data shown in Fig. 4A).

However, our vector system used an antibiotics selection system to enrich the transfectants and our *in vivo* observations were made in the absence of antibiotics selection. Thus, reversed clones that escaped from an initial treatment with *JARID1B* knockdown may have developed after a long period of time. Consistent with this possibility, observations on day 45 indicated that even initially-*JARID1B* knockdown vector-treated cells exhibited tumorigenicity. This suggested that some lentiviral-mediated *JARID1B* knockdown cells may have lost the transgene, leading to the development of transgene-free clones.

Similarly, inoculating 10^3^ cells initially showed reduced tumorigenicity on day 30, although tumor growth was observed on day 45. These results indicated that, although *JARID1B* inhibition may be a candidate molecular target for cancer therapy, a continuous inhibition system would be necessary to achieve eradication of therapy-resistant esophageal cancer.

## Discussion

In general, methylation and demethylation of histones turns genes ‘off’ and ‘on’ either by loosening their tails, which allows transcriptional factors to access DNA, or by reversing this access. Dysregulation of these activities are hallmarks of cancer through genetic and epigenetic alterations (12,13).

It was recently observed that Jarid1a/b-mediated demethylation of histone H3K4 contributed to silencing retinoblastoma target genes in senescent cells, presumably through closing the chromatin in which the silencing of retinoblastoma trigger genes was involved (17). Thus, distinct senescence-associated changes in histone-modification patterns are consistent with a repressive chromatin environment in the retinoblastoma tumor suppressor pathway (17). The results of the present study indicated that *JARID1B* knockdown (i.e., inhibition of H3K4 demethylation) resulted in the suppression of tumor cell growth *in vitro* and *in vivo*. This suggests that *JARID1B* is involved in regulating tumor cell growth in the human esophagus and is in agreement with findings of a previous report on melanoma (11).

Among retinoblastoma-mediated genes, tumor suppressor *p16/INK4A* is well documented as being involved with a senescence-associated phenotype (18). *p16/INK4A*-mediated senescence occurs through the retinoblastoma-inhibiting action of cyclin-dependent kinases and leads to G1 cell cycle arrest (18) through the interplay between their pathways and reactive oxygen species (ROS) (19). Our study indicated that exposure to hydrogen peroxide (a typical inducer of ROS) did not result in any apparent induction of a senescence-associated phenotype in esophageal squamous cell carcinoma cells that lacked tumor suppressor *p16/INK4A* in the retinoblastoma pathway. This suggests a role for *p16/INK4A* in inducing a senescence-associated phenotype with *JARID1B* inhibition (data not shown).

Therapeutic approaches for esophageal cancer include conventional treatments, such as surgical removal and chemoradiation treatment as well as gene therapy strategies, such as the introduction of the tumor suppressor *p16/INK4A* (20), expression of *IL-2*, *IL-6* and *GM-CSF* gene products (21,22), and the transduction of the herpes simplex virus-thymidine kinase gene (23,24). To achieve continuous knockdown of *JARID1B*, options include antisense oligonucleotides or low molecular therapeutic pharmacology (25). As an example, a combination of introducing the tumor suppressor gene *p16/INK4A* as gene therapy with anti-*JARID1B* treatment potentially leads to the efficient induction of a senescence-associated phenotype in esophageal cancer. This combination therapy would be efficient for eradicating therapy-resistant cancer cells, which survive after conventional treatment such as surgery, chemotherapy and radiation therapy.
